# Trefoil factor 3 mediates resistance to apoptosis in colon carcinoma cells by a regulatory RNA axis

**DOI:** 10.1038/cddis.2017.84

**Published:** 2017-03-09

**Authors:** Carlos Hanisch, Jutta Sharbati, Barbara Kutz-Lohroff, Otmar Huber, Ralf Einspanier, Soroush Sharbati

**Affiliations:** 1 Department of Veterinary Medicine, Institute of Veterinary Biochemistry, Freie Universität Berlin, Oertzenweg 19b, Berlin 14163, Germany; 2 Lise Meitner School of Science, Rudower Strasse. 184, Berlin 12351, Germany; 3 Institute of Biochemistry II, Jena University Hospital, Nonnenplan 2, Jena 07743, Germany

Trefoil factor family members (TFF1−3) are small secretory peptides on mucosal surfaces that regulate epithelial restitution and wound healing by promoting cell migration and antagonising apoptosis. They have, in common, a structural trefoil motif that is stabilised by three disulphide bonds. TFFs are often co-secreted with mucins by mucin-producing cells, helping to maintain epithelial integrity in normal tissue.^1^ TFF3 is constitutively expressed in the mammalian gastrointestinal tract; however, upregulation of TFF3 expression frequently observed in human colorectal tumour cells exerts pro-invasive activity.^2^ Like other TFFs, TFF3 is regarded as a scatter factor, which interferes with AKT, EGFR and Wnt/*β*-catenin signalling.^3^ There is increasing evidence that these pathways take an active part in progression of cancer ^4^ involving non-coding RNAs (ncRNAs).

Only a few percent of the mammalian genome are dedicated to protein synthesis, but the remaining non-coding genome either harbours important regulatory regions or encodes a plethora of ncRNAs. The research on ncRNAs has increased tremendously during the last two decades, revealing control of central cellular processes such as proliferation or apoptosis in development and disease such as gastrointestinal malignancies.^5^ Research on microRNAs unveiled many unresolved regulatory mechanisms, and it is more than likely that RNAseq-based discovery and annotation of long non-coding RNAs (lncRNAs) will accelerate this progress in the future. Along these lines, a further level of regulatory RNA networks governing cellular processes has been shown to exist by interactions between microRNAs and lncRNAs or pseudogenes.^6^

Bearing this in mind, and because many TFF3-relevant signalling pathways have been reported to be controlled by regulatory ncRNAs, we hypothesised that TFF3 may involve regulatory networks of ncRNAs to exert its effects as a scattering factor. In this context, in a recent study ^7^ we reported that TFF3 exerts resistance to TNF-*α* /IFN-*γ*-triggered apoptosis in HT-29/B6 colorectal cancer cells by a signalling axis including ncRNAs. Based on specific RT-qPCR experiments, we identified several microRNAs and lncRNAs that were differentially regulated by the expression of TFF3. Among others, TFF3 reduces the expression of miR-491-5p in a PI3K/AKT-dependent fashion, which in turn is able to interact with and downregulate the lncRNA PRINS. The miR-491-5p/PRINS-axis was shown to act as a master regulator of apoptosis sensitivity in HT-29/B6 cells. PRINS detection by fluorescence *in situ* hybridisation (FISH) revealed certain focal signals in nuclei of HT-29/B6 cells. In search for targets or interaction partners of PRINS, gain-of-function as well as loss-of-function experiments were performed, showing that the cellular concentration of PMAIP1 (NOXA) depends on PRINS availability. PMAIP1 is a pro-apoptotic protein known to recruit MCL1 to mitochondria, causing its degradation. Interestingly enough, in colorectal tumours PMAIP1 was detected both in the cytosol and in the nuclei.^8^ Finally, combined immunofluorescence and FISH experiments showed co-localised PRINS with PMAIP1 in nuclei of HT-29/B6 cells. Pull-down of PMAIP1 and co-precipitation of PRINS proved the interaction of both molecules, prompting their cooperative function in the nucleus. PRINS interacts either directly or together with ribonucleoprotein complexes with PMAIP1. Experimental reduction of PRINS causes PMAIP1 accumulation, suggesting that PRINS acts as a decoy to retain PMAIP1 in the nucleus, thereby prohibiting its pro-apoptotic activity in the cytoplasm. Our ongoing studies are further resolving the nuclear function of PMAIP1.

As mentioned above, TFF3 exerts its effects in a PI3K/AKT-dependent fashion. Currently, our knowledge about receptors for TFF peptides and how they trigger specific signalling is limited. Previously, CXCR4 has been described as a low-affinity TFF2 receptor.^9^ A more recent study reported that CXCR4/7 heterodimers induce TFF3-dependent cellular migration but not proliferation.^10^ Considering CXCR4 as a potential TFF3 receptor, diverse signalling pathways may contribute to miR-491-5p/PRINS/PMAIP1-mediated regulation of apoptosis. Upon ligand binding CXCR4 may signal through the PI3K-PDK1 cascade, finally activating P70-S6K directly or via AKT (Figure 1). In our study, recently published in *Cell Death Discovery*,^7^ we could show that chemical inhibition of AKT resulted in increased expression of miR-491-5p. On the other hand, TFF3 overexpression did not change the phosphorylation states of PDK1 and AKT, but changed P70-S6K. Otherwise, CXCR4 activation in metastatic tumour cells was reported to activate small Rho-GTPases such as CDC42.^11^ In this context, TFF3-CXCR4-mediated activation of small Rho-GTPases could have an effect on the observed regulatory axis by PAK1-dependent activation of MAPK.^12^ Moreover, since in our study TFF3 also influenced cellular levels of PLCG2, a potential role of CXCR4-dependent PLCG/PKC signalling has to be taken into account (Figure 1). Furthermore, silencing of CXCR7 was shown to induce apoptosis in a *β*-arrestin- and ERK-dependent fashion.^13^ However, whether *β*-arrestin and ERK pathway are involved in TFF3-dependent regulation of apoptosis in colon cancer progression and metastasis has still to be addressed.

In conclusion, our study^7^ provides further evidence that networks of ncRNAs together with involved ribonucleoprotein complexes present a wealth of unprecedented mechanisms that persistently revolutionise our understanding of important cellular processes, such as apoptosis and autophagy. Moreover, translational research on ncRNAs will provide targets for novel therapeutic strategies to combat, for example, resistance of malignancies to TRAIL-directed therapies or multi-drug-resistant bacterial infections.

## Figures and Tables

**Figure 1:**
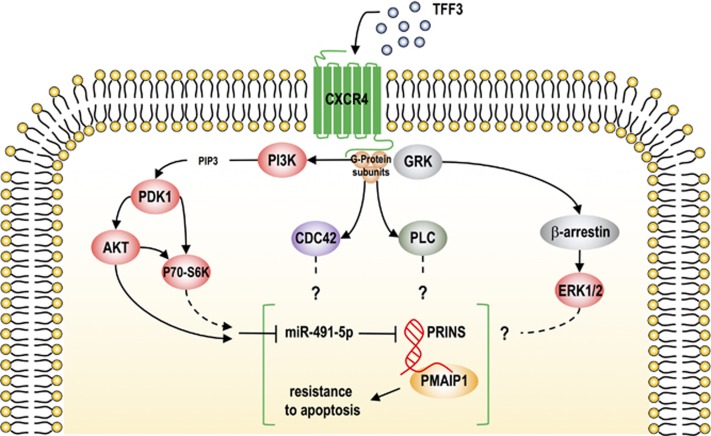
Model of potential TFF3-induced signalling pathways mediating miR-491-5p/PRINS-axis. Based on our results and data from literature the following modes of TFF3 action can be postulated. Potential binding of TFF3 to CXCR4 is considered to signal through PI3K/AKT, small Rho-GTPases, PLCG/PKC or *β*-arrestin-ERK. While our studies have demonstrated PI3K/AKT to act via miR-491-5p/PRINS to trigger apoptosis, other CXCR4-dependent signalling cascades remain to be explored

## References

[bib1] Scholven J et al Cell Physiol Biochem 2009; 23: 143–156. 19255509 10.1159/000204103

[bib2] Rivat C et al Cancer Res 2005; 65: 195–202. 15665295

[bib3] Rodrigues S et al Oncogene 2003; 22: 4488–4497. 12881705 10.1038/sj.onc.1206685

[bib4] Hu T, Li C Mol Cancer 2010; 9: 236. 20828404 10.1186/1476-4598-9-236 PMC2944186

[bib5] Jiang HJ, Wang S, Ding Y Am J Stem Cells 2014; 3: 63–73. 25232506 PMC4163605

[bib6] Salmena L et al Cell 2011; 146: 353–358. 21802130 10.1016/j.cell.2011.07.014 PMC3235919

[bib7] Hanisch C et al Cell Death Discov 2017; 3: 16106. 28149533 10.1038/cddiscovery.2016.106 PMC5279457

[bib8] Jansson AK et al Oncogene 2003; 22: 4675–4678. 12879012 10.1038/sj.onc.1206655

[bib9] Dubeykovskaya Z et al J Biol Chem 2009; 284: 3650–3662. 19064997 10.1074/jbc.M804935200 PMC2635042

[bib10] Dieckow J et al Invest Ophthalmol Vis Sci 2016; 57: 56–65. 26780310 10.1167/iovs.15-18129

[bib11] Gassmann P et al Neoplasia 2009; 11: 651–661. 19568410 10.1593/neo.09272 PMC2697351

[bib12] Qing H et al Tumour Biol 2012; 33: 985–994. 22252525 10.1007/s13277-012-0327-1

[bib13] Li XX et al Int J Oncol 2014; 45: 1649–1657. 25051350 10.3892/ijo.2014.2547

